# Well-Circumscribed Localized-Rhinophyma as a Very Rare Presentation of Rhinophyma 

**Published:** 2019-09

**Authors:** Hossein Kavoussi, Mazaher Ramezani, Fatemeh Ahmadaghaei, Iraj Ghorbani, Hamid Eftekhari Pirouz, Reza Kavoussi

**Affiliations:** 1 *Department of Dermatology, * *Hajdaie * *Dermatolgy Clinic, Kermanshah University of Medical Sciences* *, Kermanshah, Iran.*; 2 *Department* *of* *Pathology, Imam Reza Hospital, Kermanshah University of Medical Sciences, Kermanshah, Iran.*; 3 *Medical student, Kermanshah University of Medical Sciences, Kermanshah, Iran.*

**Keywords:** CO2 laser, Rhinophyma, Rosacea

## Abstract

**Introduction:**

Rhinophyma is an uncommon subtype of rosacea, the clinical diagnosis of which is straightforward. However, localized, especially well-circumscribed, rhinophyma is a very rare condition, which requires a paraclinical assessment to be accurately diagnosed.

**Case Report:**

We report a 48-year-old male patient who presented with a well-circumscribed and dark red tumoral mass of 28 mm in diameter and smooth consistency in the right nasal ala. The patient had no former and concomitant characteristic skin lesions on the other part of his face. Histopathology and immunohistochemistry assessments documented the diagnosis of rhinophyma.

**Conclusion:**

To the best of our knowledge, this is the first case report of well-circumscribed localized rhinophyma. This lesion can be treated by CO_2_ laser in a fast and efficient manner with esthetically satisfactory outcome and no significant complications.

## Introduction

Rhinophyma is the most common presentation of phymatous rosacea that mostly occurs in the nose of men. Patients with rhinophyma often have former and concomitant clinical features of rosacea, such as papule, pustule, erythema, and telangiectasia, or it may originally occur without any previous skin lesions (1,2). 

Rhinophyma is clinically characterized by a large, erythematous, and bulbous nose with dilated pores, telangiectasia, and soft, fleshy, and nodular growth (2,3). The histopathologic findings of rhinophyma are nonspecific and include sebaceous gland hyperplasia, follicular plugging with the presence of Demodex folliculorum, dilatation and enlargement of pilosebaceous structures, and varying degrees of folliculitis and granuloma formation (3). 

Localized rhinophyma is an unusual form of rhinophyma (1). Well-circumscribed localized rhinophyma (WCLR) is a very rare condition; accordingly, its difficulty in diagnosis, cosmetic problems, and selection of appropriate treatment are issues of significant importance that should be considered. 

Treatment of rhinophyma includes oral isotretinoin, cryosurgery, dermabrasion, surgical excision, and laser therapy with variable therapeutic outcomes (4-8). Herein, we reported the first case of WCLR without any former skin signs that was successfully treated by CO_2_ laser with excellent cosmetic outcomes. 

## Case Report

**Fig 1:**
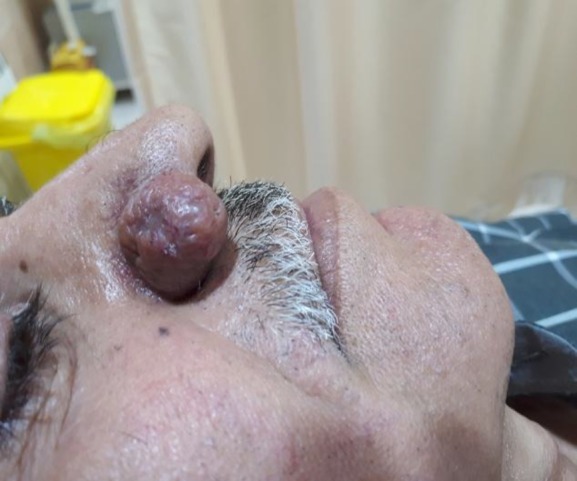
A male patient with tumor mass in nasal ala

A 48-year-old heavy smoker male driver with Fitzpatrick skin type III referred to our clinic with a tumoral mass in the right nasal ala having appeared for 18 months (Fig.1).

 In his past medical history, he had no skin lesions or systemic problems. Physical examination revealed a well-circumscribed, dark red, smooth, and consistent tumoral mass with dilated pore and sparse telangiectasias of 28 mm in diameter in the right nasal ala (Fig.1). There were no abnormal skin findings, such as redness, papulopustular lesions, and telangiectasia in other parts of the face. 

**Fig 2:**
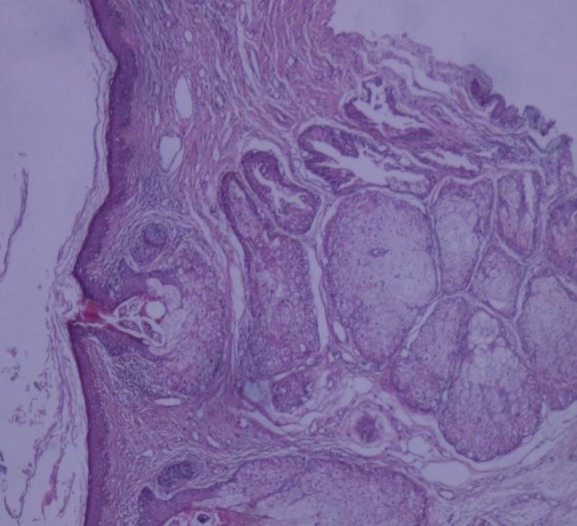
Telangiectasia, sebaceous gland hyperplasia, and perivascular infiltrate of lymphocytes with a few plasma cells and Demodex mites (Hematoxylin-Eosin stain; ×40 magnification)

The histopathologic findings of the skin lesion biopsy, performed twice over a year, were nonspecific. However, histopathological assessment of the incisional skin biopsy revealed the telangiectasia of superficial dermal vessels with perivascular infiltrate of lymphocytes and a few plasma cells. Sebaceous gland hyperplasia, perifolliculitis, and Demodex mites in pilosebaceous follicles were also noticeable (Fig.2).

Based on the results of immuno-histochemistry staining, S-100 and vimentin were positive and mild positive, respectively. Furthermore, CD68 was positive sparsely interstitially. The diagnosis of rhinophyma was established according to the findings of clinical, histopathologic, and immuno-histochemical staining. Thereafter, the patient was informed about the disease and suggested CO_2_ laser treatment for the ablation of the lesion. Before laser therapy, a horizontal section biopsy of the whole lesion was performed to rule out the superimposition of malignancy on the rhinophyma (9). 

**Fig 3:**
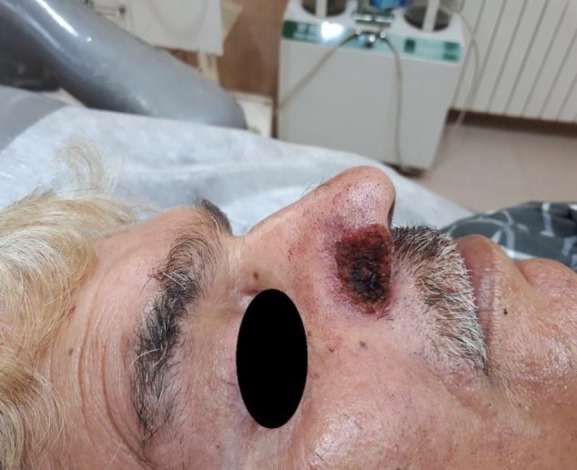
Lesion subjected to CO_2_ laser therapy leading to the alignment of the treated area with the normal surrounding skin

After obtaining written informed consent, the local injection of lidocaine with epinephrine was used as anesthesia. The lesion was subjected to CO_2_ laser therapy with continuous mode and power of 3 W. Several passes of laser therapy were performed until the treated area was in alignment with the normal surrounding skin. Between laser passes, the debris tissue was removed by a sharp disposable curettage (Fig.3). 

**Fig 4:**
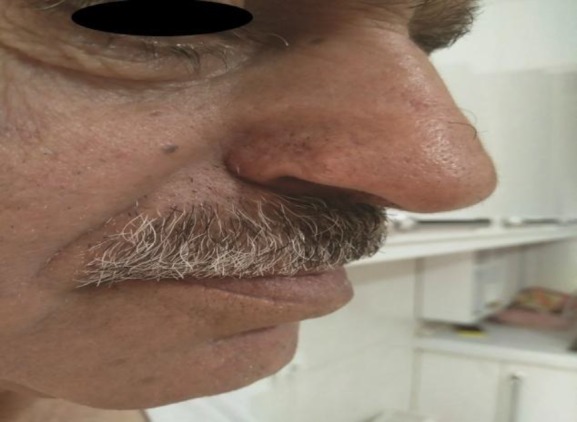
Treated area showing significant improvement with acceptable cosmetic outcomes after 6 months

For secondary intention, the induced defect was washed with Rivanol 1/1000, repair cream was applied, and the defect was dressed for 10-14 days. The patient was followed up for 6 months and showed the complete removal of the lesion with an excellent cosmetic outcome (Fig. 4).

## Discussion

We report the first case of WCLR in a middle-aged man with Fitzpatrick skin type III without any previous skin lesions. The lesion was successfully ablated by CO_2_ laser with an excellent cosmetic outcome. According to multiple studies, rhinophyma is an end-stage and severe form of rosacea, and most of the patients have previous skin lesions. Localized rhinophyma is a subtype and an unusual presentation of rhinophyma that is associated with difficulty in diagnosis and cosmetic problem (1,2).

The clinical diagnosis of typical rhinophyma is straightforward. However, unlike the typical rhinophyma, definite clinical diagnosis of localized rhinophyma and WCLR is not possible and requires histopathologic and immunohistochemical evaluations. In the differential diagnosis of tumors, especially malignant skin tumors like squamous cell carcinoma, basal cell carcinoma, and sebaceous carcinoma should be considered(1-3).

Early diagnosis of WCLR is very important because it leads to the avoidance of further paraclinical assessment, suggestion of appropriate treatment, and reduction of emotional stress and cost in the patients. The CO_2_ laser is a popular modality in most outpatient surgery centers. This approach is suitable for the treatment of some malignant and most of pre-malignant and benign skin lesions, including rhinophyma (10).

In a number of studies, patients with rhinophyma were subjected to CO_2_ laser therapy, and most of them showed acceptable improvement (4-6). Nonetheless, the limitations of these studies are the need for multiple sessions and time-consuming nature of the procedure with variable outcomes.

In some studies, CO_2_ laser, along with other laser modalities, such as pulsed dye and erbium-YAG laser, was used for the ablation of rhinophyma (7,8). However, we think that this combination therapy is time-consuming and costly; moreover, other lasers, except for CO_2_ laser, are not accessible in most outpatient clinics. 

Madan et al. treated 124 patients with rhinophyma by high-power continuous cutting mode of CO_2_ laser (8). They concluded CO_2_ laser as an effective treatment with a low risk of side‐effects and high patient satisfaction. Our patient was subjected to low-power (3 W) CO_2_ laser therapy using continuous cutting mode and a sharp and disposable curettage as adjuvant therapy for the removal of debris between laser passes.

In our method, the use of low-power CO_2_ laser therapy and curettage as adjuvant therapy was associated with low thermal damage to the underlying and surrounding tissues of the treated area. These facilities provide easy application, few sessions, and short-term procedure with a low possibility of side effects, as well as inexpensive and acceptable cosmetic and treatment outcomes.

## Conclusion

To the best of our knowledge, this is the first case report of WCLR. Our procedure by CO_2 _laser was found to be an easy, rapid, and effective treatment of rhinophyma leading to an excellent cosmetic outcome with low risk or few complications.
